# Supplementary material for: Identification of Potential Mechanisms of Rk1 Combination with Rg5 in the Treatment of Type II Diabetes Mellitus by Integrating Network Pharmacology and Experimental Validation
Source: Int J Mol Sci. 2023 Oct 2;24(19):14828. doi: 10.3390/ijms241914828 (PMC10573417; doi:10.3390/ijms241914828)
Supplement: Supplementary file 1 [file ijms-24-14828-s001.zip › ijms-2626220-supplementary.pdf]

Supporting Information

Identification of Potential Mechanisms of Rk1 Combination with Rg5 in the Treatment of Type II Diabetes Mellitus by Integrating Network Pharmacology and Experimental Validation

Yao Liu, Jingjing Zhang, Chao An, Chen Liu, Qiwen Zhang, Hao Ding, Saijian Ma and Wenjiao Xue *

Shaanxi Key Laboratory of Qinling Ecological Security, Shaanxi Institute of Microbiology, Xiying Road 76, Xi'an 710043, China; liuyao181002@163.com (Y.L.); zjj_1712@163.com (J.Z.); anchor0216@sina.com (C.A.); 13892806669@163.com (C.L.); 13772195921@163.com (Q.Z.); dhwj2010@126.com (H.D.); masaijian@163.com (S.M.)

* Correspondence: x-wenjiao@163.com

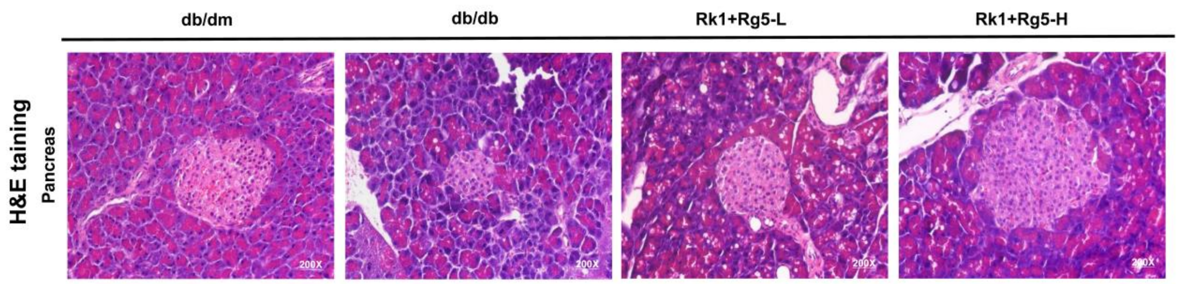

Figure S1. H&E staining of pancreas tissues in db/db mice.

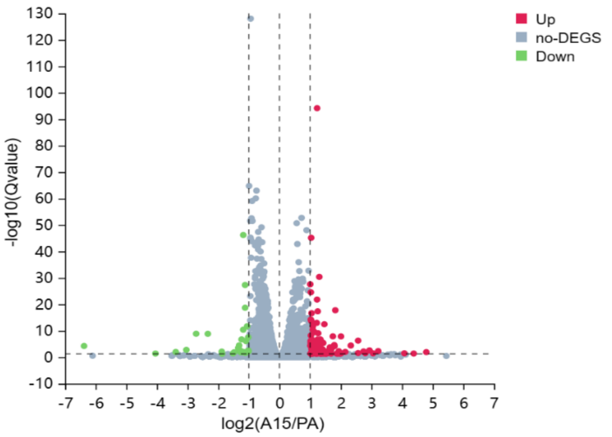

Figure S2. Volcano map of differential genes between Rk1+Rg5 treatment group and PA group.

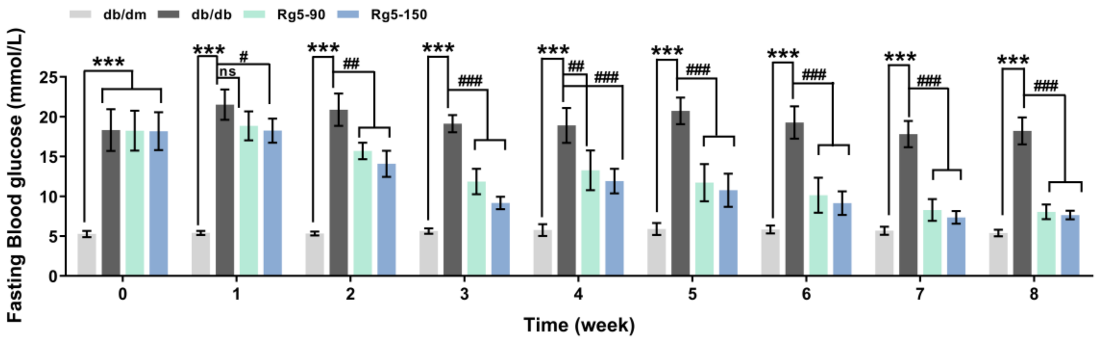

Figure S3. Effect of ginsenoside Rg5 on fasting blood glucose in db/db mice.
